# The Arc of Astrocyte Aging: Insights From scRNAseq

**DOI:** 10.1093/geroni/igab046.1437

**Published:** 2021-12-17

**Authors:** Margarita Meer, Christopher Minteer, Morgan Levine

**Affiliations:** 1 Yale School of Medicine, Yale University, Connecticut, United States; 2 Yale University, Yale University, Connecticut, United States; 3 Yale University, New Haven, Connecticut, United States

## Abstract

There is an urgent need to increase our understanding of brain aging and its role in neurodegeneration. While, evidence suggest that many hallmarks of aging, including epigenetic alterations and cellular senescence may be implicated in dementia, studying these and other progressive molecular changes in the brain remains extremely challenging. We asked whether something as simple as artificially aging cells in culture could recapitulate the changes that occur during organismal aging. To test this, we passaged human primary astrocytes and performed single-cell RNA sequencing (scRNAseq) of cells at passages 2-10. We observe that the sequential passaging—that terminates with a cluster of senescent cells—can be captured by manifolds and used to quantify a pseudo-time measure of progressive transcriptional changes. We identify genes underlying this transition and apply this signature of in vitro astrocyte passaging to scRNAseq from human and mouse brain aging studies, demonstrating associations with aging and neuropathology.

